# Moving Standard Deviation of Trunk Acceleration as a Quantification Index for Physical Activities: Validation Study

**DOI:** 10.2196/63064

**Published:** 2025-04-08

**Authors:** Takuya Suzuki, Yuji Kono, Takayuki Ogasawara, Masahiko Mukaino, Yasushi Aoshima, Shotaro Furuzawa, Yurie Fujita, Hirotaka Matsuura, Masumi Yamaguchi, Shingo Tsukada, Yohei Otaka

**Affiliations:** 1 Department of Rehabilitation, Fujita Health University Hospital, Toyoake, Japan; 2 NTT Basic Research Laboratories and Bio-medical Informatics Research Center, NTT Corporation, Atsugi, Japan; 3 Department of Rehabilitation Medicine, Fujita Health University School of Medicine, Toyoake, Japan; 4 Department of Rehabilitation Medicine, Hokkaido University Hospital, Kita14 Nishi5, Kita-Ku, Sapporo, 0608648, Japan, 81 11-706-6066

**Keywords:** smart clothing, step count, moving standard deviation of acceleration, MSDA, wheelchair, activity quantification, physical activities, validation study, accelerometer, regular gait patterns, older people, aging, motor impairments, step detection, stroke, hemiparesis, measurement system, walking, mobility, rehabilitation

## Abstract

**Background:**

Step count is used to quantify activity in individuals using accelerometers. However, challenges such as difficulty in detecting steps during slow or irregular gait patterns and the inability to apply this method to wheelchair (WC) users limit the broader utility of accelerometers. Alternative device-specific measures of physical activity exist, but their specificity limits cross-applicability between different device sensors. Moving standard deviation of acceleration (MSDA), obtained from truncal acceleration measurements, is proposed as another alternative variable to quantify physical activity in patients.

**Objective:**

This study aimed to evaluate the validity and feasibility of MSDA for quantifying physical activity in patients with stroke-induced hemiparesis by comparing it with the traditional step count.

**Methods:**

We enrolled 197 consecutive patients with stroke hemiparesis admitted to a convalescent rehabilitation ward. Using the hitoe system, a smart clothing–based physical activity measurement system, we measured the MSDA of trunk movement and step count. The correlation between MSDA and step count was examined in all participants. Based on their daily living mobility levels, measured using the Functional Independence Measure (FIM), participants were categorized into 6 subgroups: FIM1-4, FIM5 (WC), FIM5 (walking), FIM6 (WC), FIM6 (walking), and FIM7 (walking). Intersubgroup differences in MSDA were analyzed.

**Results:**

A strong correlation was observed between MSDA and step count (*r*=0.78; *P*<.001), with a stronger correlation in the walking group (*r*=0.79; *P*<.001) compared with the WC group (*r*=0.55; *P*<.001). The Shapiro-Wilk test indicated no significant results for MSDA across all subgroups, supporting a normal distribution within these groups. In contrast, the step count data for the WC subgroups showed significant results, indicating a deviation from a normal distribution. Additionally, 10.2% (20/197) of participants recorded zero steps, demonstrating a floor effect in the step count data. The median MSDA values for the 6 subgroups (FIM1-4, FIM5 WC, FIM5 walking, FIM6 WC, FIM6 walking, and FIM7) were 0.006, 0.007, 0.010, 0.011, 0.011, and 0.014, respectively, reflecting their levels of independence based on the FIM mobility scores. The median step counts for these subgroups were 68, 233, 1386, 367, 2835, and 4462, respectively. FIM5 participants who walked had higher step counts than FIM6 participants using WCs, though the difference was marginally but not statistically significant (*P*=.07), highlighting the impact of mobility type (walking vs WC).

**Conclusions:**

The results suggest the validity of MSDA as a variable for physical activity in patients with stroke, applicable to patients with stroke irrespective of their mobility measures. This finding highlights the potential of MSDA for use in individuals with motor impairments, including WC users, underscoring its broad utility in rehabilitation clinical practice.

## Introduction

Poststroke rehabilitation is primarily undertaken to enhance patient independence in activities of daily living (ADL). To achieve this goal, improving movement ability and exercise tolerance is important, which is directly reflected in the daily amount of activity. Increases in physical activity and extended rehabilitation time are associated with improved physical function and shorter hospital stays [1,2]. Higher levels of physical activity can decrease the mortality risk [3]. Therefore, physical activity requirements should be actively assessed in patients in the poststroke stage. To quantify physical activity, the number of steps, measured using an accelerometer, is widely used and is a valid and reliable variable for assessing physical activity in clinical rehabilitation settings [4,5]. Numerous studies have established a close association between the number of steps taken and health-related quality of life [6], self-efficacy [7], and the risk of stroke recurrence [8]. Accelerometers are reliable and validated devices that can objectively measure physical activity in patients with stroke [9]. Exercise therapy, when supplemented with accelerometer feedback and a goal-centered step-activity monitoring program, can enhance the physical activity of hospitalized patients recovering from ischemic stroke [10,11].

While widely used to quantify activity, counting steps is only applicable to ambulatory patients. Most of the previous studies have focused on patients with stroke who could walk independently. However, a substantial number of these patients face challenges with walking in their daily lives. During discharge from acute hospitalization, approximately 30%‐50% of patients had a modified Rankin scale score of ≥4 [12], indicating significant mobility impairment. While step count, an acceleration threshold–based measure of activity, may partially capture movement in everyday activities, its validity in accurately measuring the activity of patients with walking difficulties remains questionable. These considerations highlight the need for acceleration-based measures that can be effectively used in patients with difficulty walking.

To address this issue, we proposed an alternative metric: the moving standard deviation of acceleration (MSDA), which is derived from the SD of the acceleration norm that is measured from the trunk using an accelerometer placed on the chest. The chest placement of the accelerometer enables movement measurement in a seated position, which is expected to have advantages in activity monitoring in wheelchair (WC) users. The utility of the MSDA as an activity-quantification measure has been validated previously, showing a strong correlation between the MSDA and percentage oxygen uptake reserve in healthy participants and between MSDA and percentage heart rate reserve in patients with motor dysfunction due to cerebrovascular disease [13]. However, its validity compared with traditional accelerometry-based measures, such as step count, remains unexplored. Additionally, the potential advantages of MSDA in quantifying physical activity for individuals with limited mobility, including WC users, have yet to be confirmed with real-world data.

Consequently, this study focused on investigating the validity and feasibility of the MSDA measurement with a hitoe system in the measurement of physical activity in patients with stroke, with or without a WC. Specifically, our objectives were (1) to examine the correlation between the MSDA and the number of steps taken and (2) to compare their data characteristics, which includes an understanding of the features of data distribution and investigating their respective relationships with the level of mobility limitation.

## Methods

### Participants

Between January 2018 and February 2020, we enrolled consecutive patients with stroke hemiparesis who were admitted to the recovery rehabilitation ward of Fujita Health University Hospital. The inclusion criteria were (1) a confirmed diagnosis of stroke resulting in hemiparesis, (2) aged 18 years or older, and (3) the provision of written informed consent. The exclusion criteria were (1) noncompliance with wearing the hitoe measurement wear and (2) the presence of severe comorbid conditions, such as advanced heart failure or respiratory diseases requiring oxygen support that could interfere with participation in the rehabilitation program or data collection.

### Study Design and Protocol

This single-center cross-sectional study comprised participants who underwent physical activity assessment within 1 week of admission to the recovery rehabilitation ward.

### Ethical Considerations

This study was conducted in accordance with the ethical principles outlined in the Declaration of Helsinki and was approved by the Institutional Review Board of Fujita Health University (approval number HM17-220). All eligible patients who were admitted were informed about the study by the clinicians and provided with detailed information about its objectives and procedures. Those who agreed to participate were enrolled in the study after providing written informed consent. To ensure privacy, all data were deidentified before analysis. Participants did not receive financial compensation for their involvement in this study.

### Measurement

A hitoe system (NTT Corporation and Toray Industry, Inc) was used to measure the amount of physical activity [14]; this study specifically evaluated its accelerometry-based physical activity measurement function. The hitoe system is a clothing-based activity-monitoring system that consists of clothing with embedded electrodes for heart rate measurement, a transmitter equipped with an embedded accelerometer, a data receiver (which can be a smartphone or an Internet of Things gateway), and a data server. The wearer’s activity is measured using an accelerometer to capture physical movement and a heart rate monitor to assess load. The transmitter was securely fastened to the chest with a tight-fitting strap to minimize measurement noise. The noninferiority of accuracy in accelerometry-based measurements using the hitoe system has been demonstrated previously through comparisons with various accelerometry-based devices, including waist-worn accelerometers (pedometers) [15]. The sampling rate was set to 25 Hz. Monitoring started before noon on the first assessment day and ended in the afternoon on the third assessment day. As clothing was used for monitoring, monitoring was paused during bathing. After bathing, the hitoe wear was changed, and the monitoring resumed. To mitigate the impact of daily fluctuations and compensate for deficiencies [16], we used 48-hour data to calculate the 24-hour ensemble average. Ensemble averaging is a method in which multiple measurements recorded at the same time of the day are averaged together. For instance, the data recorded between 5 PM and 5:01 PM on the first day was averaged with the data recorded at the same time on the second day, producing the ensemble average for 5 PM-5:01 PM. This process was repeated for all time points throughout the 24-hour period. The data with ≥5% measurement error after ensemble averaging were excluded from the analysis. The acquired 24-hour average values of minute-by-minute data on the number of daily steps and the MSDA were used for the analysis. The step-detection capability of this system has been validated using data from various commercial pedometers [15]. The MSDA was derived from a 2-second window of 50 samples of the norm of truncal acceleration, as measured by the accelerometer. The acceleration norm was computed using the following equation:


$$\text{Norm}=\sqrt{\left(x^{2}+y^{2}+z^{2}\right)}$$

where x, y, and z represent the vertical, lateral, and anterior/posterior axes, respectively.

Mobility levels of the patients were assessed using the Functional Independence Measure (FIM) scores. The FIM consists of 18 (13 motor and 5 cognitive) items. Each item is rated on a 7-point scale from 1 (full assistance) to 7 (independence). The data on other clinical characteristics including age, sex, height, weight, and disease were collected from the patients’ medical records.

### Data Analysis

Continuous variables are presented as mean and SD and categorical variables as percentages. The Shapiro-Wilk test was used to examine the normality of the number of steps and MSDA. To further investigate the characteristics of these distributions, their skewness and kurtosis were examined using the Fisher-Pearson coefficient of skewness (g1) and Fisher coefficient of kurtosis (g2) [17]. The relationship between MSDA and number of steps was examined using Spearman rank correlation coefficient. This analysis was conducted for all participants and separately for the two groups: those in the walking group and those in the WC group. Finally, we categorized all participants into 6 subgroups based on the mobility scores from the FIM and their mobility patterns as follows: FIM1-4, FIM5 WC, FIM5 walking, FIM6 WC, FIM6 walking, and FIM7. The Kruskal-Wallis test was performed to analyze the association between MSDA and the number of steps. The Bonferroni method was used to control for multiple comparisons.

All analyses were performed using SPSS (version 26; IBM Corp). Statistical significance was set at *P*<.05.

## Results

A total of 197 patients (129 men) were enrolled and included in the analysis (Table 1). The mean age was 66.1 (SD 12.5) years. Of these, 61 patients were ambulatory, and 136 patients used WCs. The median number of days after onset was 33 (IQR 24‐45). The average MSDA and number of steps were 0.009 (SD 0.003) and 1229 (SD 1834), respectively.

**Table 1.** Participant characteristics in the study cohort. Baseline characteristics of 197 participants including participants’ demographics, stroke etiology, mobility status, and Functional Independence Measure (FIM) scores (motor, cognitive, and total) are summarized.

Variables	Values
Age (years), mean (SD)	66.1 (12.5)
Sex, n (%)	
Male	129 (65.5)
Female	68 (34.5)
Height (cm), mean (SD)	161.7 (15.5)
Weight (kg), mean (SD)	58.6 (13.7)
Days after onset, median (IQR)	33 (24‐45)
Etiology, n (%)	
Cerebral hemorrhage	71 (36)
Cerebral infarction	94 (47.7)
Subarachnoid hemorrhage	16 (8.1)
Others	16 (8.1)
Mobility, n (%)	
Walking	61 (31)
Wheelchair	136 (69)
FIM-motor, mean (SD)	48.5 (26.0)
FIM-cognitive, mean (SD)	22.5 (10.2)
FIM-total, mean (SD)	70.7 (34.6)

**Figure 1.**
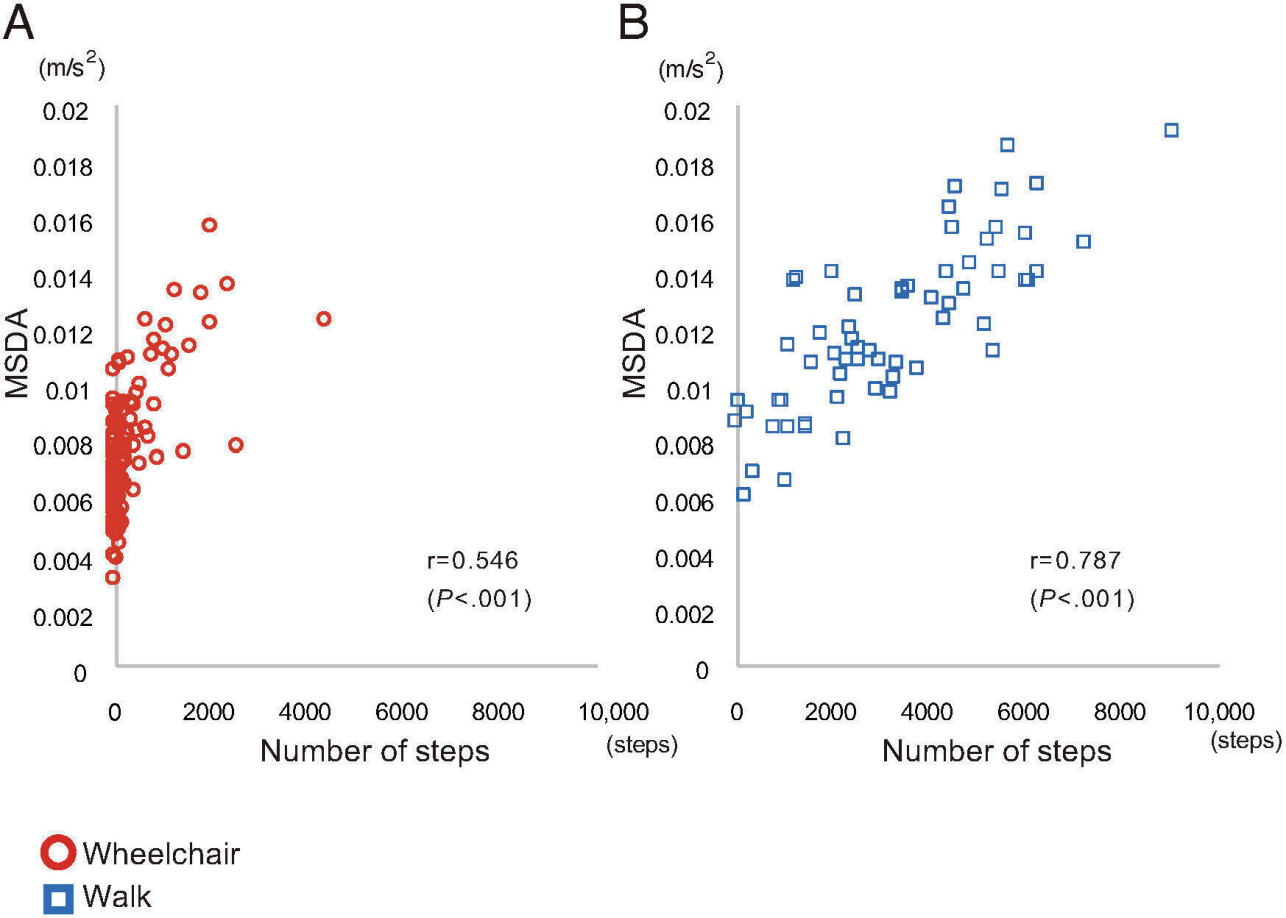
Scatter plot showing the correlations between MSDA and number of steps in the (A) wheelchair group (n=136) and (B) walking group (n=61). Open circles and open squares represent the wheelchair and walking groups, respectively. MSDA: moving standard deviation of acceleration.

The relationship between the MSDA and the number of steps is shown in Figure 1. Significant positive correlations were found for all participants (*r*=0.784; *P*<.001), the walking group (*r*=0.787; *P*<.001), and the WC group (*r*=0.546; *P*<.001). When excluding the extreme data with 0 values, the correlation coefficient was raised, for all participants (*r*=823; *P*<.001), the walking group (*r*=0.798; *P*<.001), and the WC group (*r*=0.634; *P*<.001).

**Figure 2.**
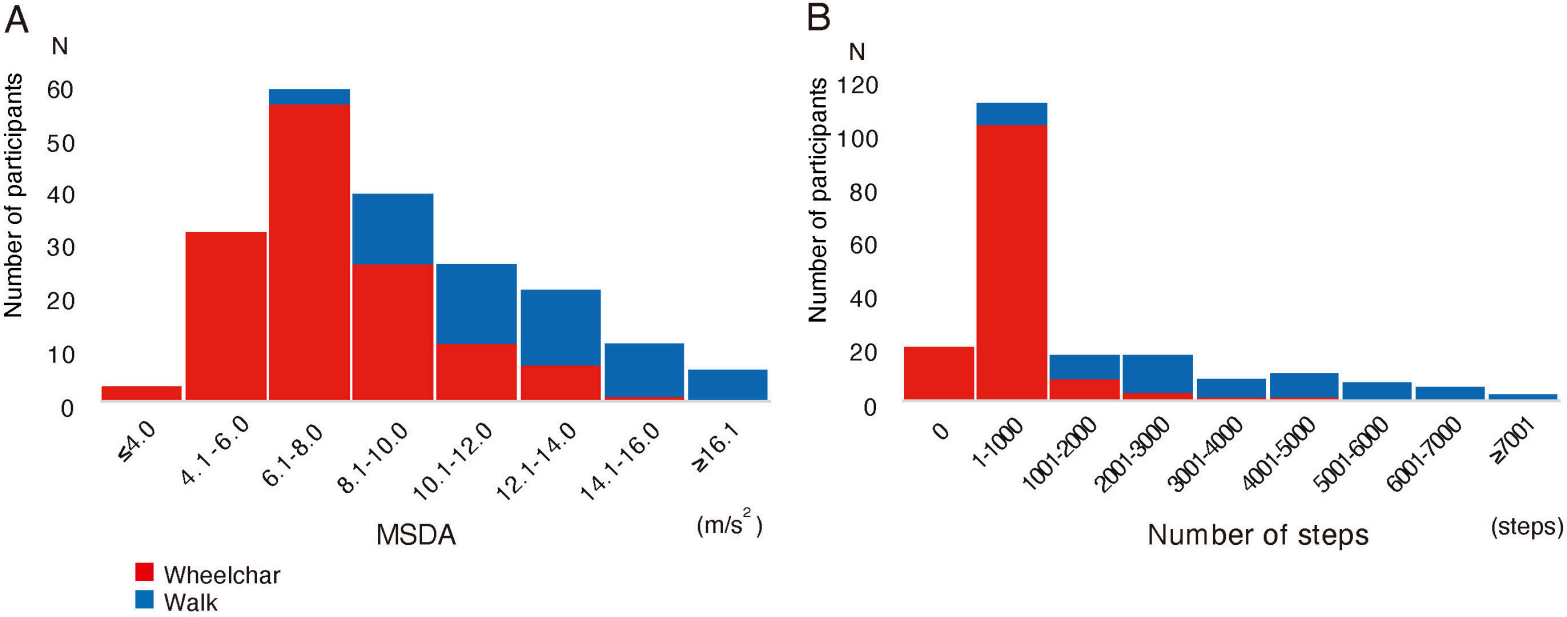
Histograms showing the distribution of (A) MSDA and (B) daily step count for all 197 participants, divided by mobility type. The wheelchair group is represented in red and the walking group in blue. MSDA: moving standard deviation of acceleration.

The detailed features of the MSDA distribution and the number of steps for all participants are shown in Figure 2. Neither the MSDA nor the number of steps exhibited a normal distribution. The distributions of MSDA and steps exhibited right-skewness, with skewness coefficients of 0.78 and 1.72, respectively (zero when normally distributed). The kurtosis coefficient for MSDA was 3.02, which is close to the typical value of 3 for a normal distribution. In contrast, the kurtosis coefficient for steps was 5.26, indicating a higher level of kurtosis. The normality of the distribution was further tested in each of the 6 subgroups (Table 2). The distribution of MSDA data within subgroups exhibited smaller deviations from a normal distribution in terms of kurtosis and skewness coefficients, suggesting that it was closer to a normal distribution compared with the steps data. The Shapiro-Wilk test did not yield significant results for MSDA in all the subgroups, whereas the step data showed significance, especially within the WC subgroups. In the step count, 10.2% (20/197) of participants exhibited zero count, showing the floor effect.

**Table 2.** Kurtosis and skewness values and results of the Shapiro-Wilk test. This table summarizes the normality test results for the moving standard deviation of acceleration (MSDA) and daily step count in participants classified by mobility type and functional levels. For normal distribution, kurtosis (g2)=3 and skewness (g1)=0.

	MSDA			Number of steps		
	Kurtosis (g2)	Skewness (g1)	*P* value (Shapiro-Wilk)	Kurtosis (g2)	Skewness (g1)	*P* value (Shapiro-Wilk)
WC^a^ 1‐4 (n=94)	3.33	0.51	.18	24.33	4.32	<.001
WC 5 (n=23)	3.15	0.94	.05	2.22	0.75	.005
WC 6 (n=19)	2.59	0.05	.92	4.37	1.38	.002
Walking 5 (n=15)	2.48	0.27	.79	1.64	0.01	.23
Walking 6 (n=18)	2.75	0.47	.67	1.95	0.09	.83
Walking 7 (n=28)	2.63	–0.38	.62	2.57	0.10	.42
WC (n=136)	3.91	0.93	<.001	19.48	3.61	<.001
Walking (n=61)	2.56	0.19	.72	2.61	0.40	.11
All participants (n=197)	3.02	0.78	<.001	5.26	1.72	<.001

a WC: wheelchair.

**Figure 3.**
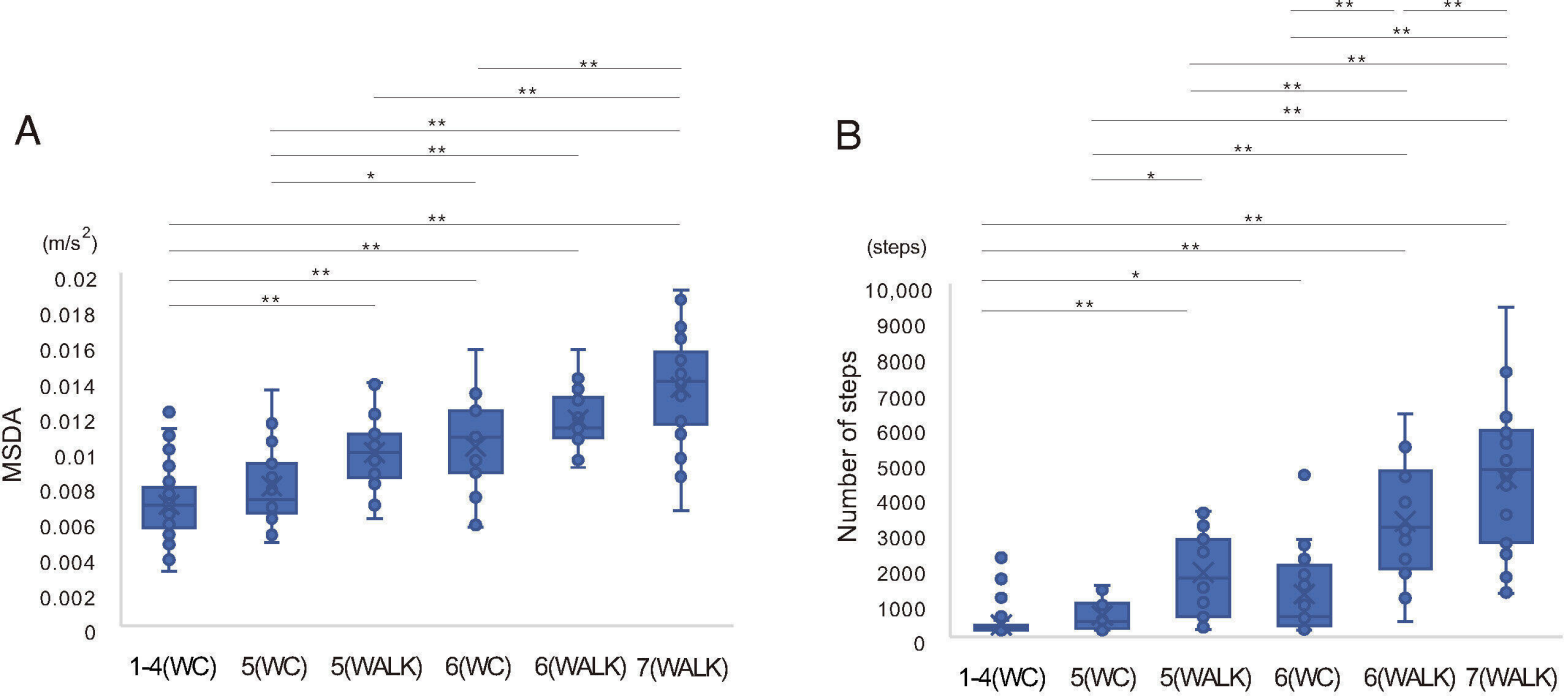
Relationship between Functional Independence Measure (FIM)-mobility scores and the MSDA for 6 groups divided by mobility type: FIM1-4, FIM5 (wheelchair), FIM5 (walking), FIM6 (wheelchair), FIM6 (walking), and FIM7 (walking). Physical activity was compared among the 6 subgroups according to the FIM scores of the mobility items and mobility patterns. (A) MSDA and (B) number of steps. MSDA: moving standard deviation of acceleration. *P<.05, **P<.01.

The relationships between the 6 subgroups according to the FIM scores of mobility items, mobility patterns, MSDA, and number of steps are shown in Figure 3. The median MSDA values in the 6 subgroups FIM1-4, FIM5 WC, FIM5 walking, FIM6 WC, FIM6 walking, and FIM7 were 0.006, 0.007, 0.010, 0.011, 0.011, and 0.014, respectively. Significant differences were found in MSDA between the low-FIM and high-FIM subgroups (Figure 3A). The median number of steps in the 6 subgroups were 68, 233, 1386, 367, 2835, and 4462, respectively. Significant differences in the steps between the low-FIM and high-FIM subgroups are shown in Figure 3B. The difference between FIM5 participants with walking and FIM6 WC was marginal (*P*=.07).

## Discussion

### Principal Findings

This study investigated the characteristics of the MSDA and step count in measuring physical activity in patients with stroke-induced hemiparesis. The MSDA not only had a positive correlation with the number of steps but was also associated with the FIM-mobility score, showing the validity as activity measurement index. Additionally, the MSDA demonstrated potential advantages in quantifying activity levels in patients with walking difficulties. First, in contrast to the step count, the distribution of the MSDA exhibited a closer alignment with a normal distribution, particularly within subgroups of patients using WCs and those with similar ADL levels. Second, the MSDA did not present a floor effect within the WC group, which was observed with the step count. Finally, the MSDA was consistent with varying levels of mobility independence, showing no significant differences across types of mobility measures (walking or WC use). In contrast, the step count was more susceptible to the mode of mobility; significant differences were observed in the step values between groups with the same level of independence but using different mobility measures.

The findings of this study demonstrated the strength of the MSDA as a measure of physical activity in patients after stroke, who often present with gait disturbance and relies on WC. The conducted analyses on the correlation between the step counts and MSDA and the distribution of those in the poststroke rehabilitation inpatients, of whom approximately 70% relied on a WC for mobility, support the usability of the MSDA in those with limited gait functionality. Our results indicated that the MSDA was positively correlated with the number of steps, which is an established measure of physical activity, supporting the validity of the MSDA. Further analysis revealed a weaker correlation in patients who used WCs. The weaker correlation between step counts and MSDA in WC users can be attributed to the skewed distribution and floor effects in their step counts. Notably, excluding participants with a step count of zero results in an improved correlation coefficient. Further stratified examination revealed that, in contrast to the number of steps, the MSDA was normally distributed within all levels of mobility independence. These findings suggest that the MSDA has broad applicability, including that for patients in poststroke stage who use WCs.

The strength of the MSDA in measuring physical activity among patients in poststroke stage, with fewer issues in distribution patterns, may stem from its nature as a continuous variable. The number of steps, which is a frequently used variable for activity monitoring, is a threshold-based variable that measures the number of movements with greater acceleration beyond a specific threshold [16]. However, this approach may induce challenges when applied to patients with neurological disorders who often manifest irregular step patterns and slower gait speeds. The use of accelerometers to count steps can produce misleading results in such cases [18]. In contrast, the MSDA quantifies truncal oscillations; as the trunk is the body’s heaviest segment, this can, without imposing a specific threshold, be captured as a continuous value [13]. Thus, the MSDA could be more sensitive to variations in nonwalking activities. Consequently, the MSDA distribution encompasses both walking and nonwalking activities; this characteristic of the MSDA underscores its potential application as a comprehensive metric for evaluating activity levels irrespective of individual mobility methods, whether walking or using WCs.

Furthermore, our results illustrate that the MSDA effectively reflects the level of independence in daily life. Previous studies have demonstrated a strong correlation between physical activity and independence levels; for instance, the Life-Space Assessment, a physical activity measure, shows a positive correlation with Lawton instrumental ADL scores [19,20]. A similar positive correlation has been noted between accelerometer-measured physical activity and FIM-motor scores in patients with stroke [21].

The study’s results further support previous observations and reveal significant differences in both the MSDA and step count between patients with and without independent mobility. Furthermore, in contrast to the step count, the MSDA appears to be less influenced by the mode of mobility. In the case of mobility levels FIM5 and FIM6, no significant differences were observed between patients using WCs and those walking, while significant disparities in step counts were observed across different mobility measures at both levels, leading to reduced coherence with respect to levels of independence—it tends to be higher for patients capable of walking with a level of FIM 5, as opposed to those who are using WC with a FIM score of 6. These findings suggest that the MSDA can serve as a universal measure of physical activity, regardless of an individual’s gait capabilities. This objective measure could be beneficial for providing feedback and setting goals for rehabilitation, to encourage daily activities. Many community-dwelling patients with a stroke fail to achieve the recommended activity level [22]. Thus, using the MSDA to track daily activities could offer a valuable strategy for promoting more active daily living in the realm of rehabilitation.

### Limitations

This study has some limitations. First, its applicability to nonhospitalized patients warrants further exploration. The study was conducted in a rehabilitation ward with a flat floor, which differs from the environment outside the hospital. With barriers such as steps or uneven surfaces, the MSDA values may be higher than those in hospital wards. The impact of environmental variables on MSDA merits investigation in future studies. Despite these considerations, this study is meaningful in demonstrating the advantage of MSDA in the measurement of physical activity in patients with low walking ability or WC users.

Second, the feasibility of using the MSDA with the hitoe system in a home setting should be further explored. In this study, therapist assistance was required for fitting and ensuring proper placement to maintain measurement quality. While the system has been designed to allow patients with hemiparesis to wear it independently, those with severe hemiparesis may still encounter difficulties. For this system to be effectively integrated into daily activity management for patients with hemiparesis, ease of fitting is essential. Further investigation is needed to adapt and implement this system in practical clinical settings.

Third, as the MSDA reflects truncal movement oscillations without identifying the specific movements involved, certain neurological symptoms may influence the measurements. For instance, patients with Parkinson disease experience tremors that can affect the MSDA values, which reflect movement variability. Further research is needed to clarify the movements or symptoms that may distort MSDA values. Finally, to enhance understanding, the clinical significance of MSDA values should be further investigated, specifically in terms of interpreting different values as corresponding levels of physical activity. While the step count serves as an easily understandable and intuitive metric for clinical feedback, the MSDA value lacks this straightforwardness, posing challenges for patients in correlating it with specific activities. Therefore, fostering a deeper comprehension and interpretation of this variable within clinical contexts is imperative. Efforts should be directed towards contextualizing the MSDA values and making them more intuitive and meaningful for patients.

### Conclusion

This study indicates that the MSDA is a valid variable for measurement of physical activity, applicable to patients with stroke irrespective of their mobility measures. Future research is warranted to examine the factors influencing MSDA values and to establish the clinical utility of this variable.

## Abbreviations

ADL: activities of daily living
FIM: Functional Independence Measure
MSDA: moving standard deviation of acceleration
WC: wheelchair
